# A Brain-Derived Neurotrophic Factor Mimetic Is Sufficient to Restore Cone Photoreceptor Visual Function in an Inherited Blindness Model

**DOI:** 10.1038/s41598-017-11513-5

**Published:** 2017-09-12

**Authors:** Conor Daly, Lisa Shine, Theresa Heffernan, Sudhakar Deeti, Alison L. Reynolds, John J. O’Connor, Eugène T. Dillon, David J. Duffy, Walter Kolch, Gerard Cagney, Breandán N. Kennedy

**Affiliations:** 1 UCD School of Biomolecular & Biomedical Science, UCD Conway Institute, University College Dublin, Belfield, Dublin 4, Ireland; 2 Food for Health Ireland, Science Centre South, University College Dublin, Belfield, Dublin 4, Ireland; 3 Systems Biology Ireland, Science Link Building, University College Dublin, Belfield, Dublin 4, Ireland; 4 School of Medicine, University College Dublin, Belfield, Dublin 4, Ireland; 5 Present Address: The Whitney Laboratory for Marine Bioscience & Sea Turtle Hospital, University of Florida, 9505 Ocean Shore Blvd. St. Augustine, FL, 32080-8610 USA

## Abstract

Controversially, histone deacetylase inhibitors (HDACi) are in clinical trial for the treatment of inherited retinal degeneration. Utilizing the zebrafish *dye* ^*ucd6*^ model, we determined if treatment with HDACi can rescue cone photoreceptor-mediated visual function. *dye* exhibit defective visual behaviour and retinal morphology including ciliary marginal zone (CMZ) cell death and decreased photoreceptor outer segment (OS) length, as well as gross morphological defects including hypopigmentation and pericardial oedema. HDACi treatment of *dye* results in significantly improved optokinetic (OKR) (~43 fold, p < 0.001) and visualmotor (VMR) (~3 fold, p < 0.05) responses. HDACi treatment rescued gross morphological defects and reduced CMZ cell death by 80%. Proteomic analysis of *dye* eye extracts suggested BDNF-TrkB and Akt signaling as mediators of HDACi rescue in our dataset. Co-treatment with the TrkB antagonist ANA-12 blocked HDACi rescue of visual function and associated Akt phosphorylation. Notably, sole treatment with a BDNF mimetic, 7,8-dihydroxyflavone hydrate, significantly rescued *dye* visual function (~58 fold increase in OKR, p < 0.001, ~3 fold increase in VMR, p < 0.05). In summary, HDACi and a BDNF mimetic are sufficient to rescue retinal cell death and visual function in a vertebrate model of inherited blindness.

## Introduction

There is an unmet clinical need to develop effective treatments for inherited vision loss. Inherited retinal degenerations (iRD) are clinically and genetically heterogeneous, presenting as diverse forms of blindness that include retinitis pigmentosa (RP), Leber congenital amaurosis (LCA), achromatopsia (ACHM), cone-rod dystrophy (CORD) and macular dystrophy (MD)^1^. iRDs are estimated to affect 1 in every 2000–3000 people^2^, and many are characterised by loss and/or dysfunction of photoreceptor cells. Potential interventions include drug-, gene- or cell-based therapy^3^. Cell therapy has potential to restore functional retinal cells irrespective of the genetic cause of iRD^4, 5^. However, the integration of replacement progentitor photoreceptor cells which restored visual function in pre-clinical models^6^ has been challenged by evidence demonstrating intracellular exchange between donor and host cells^7^. Gene therapy demonstrated initial success in clinical trials^8, 9^. However, this approach is hampered by limited applicability due to genetic heterogeneity of iRD^10^. Additional concerns regarding long-term efficacy are exemplified by RPE65 gene replacement which fails to halt the progressive loss of photoreceptors^11^. An alternative to replacing defective cells or genes is to identify neuroprotective agents preventing loss of visual function^12^. Selection of neuroprotectants can be based on efficacy in other neurodegenerations or *de novo* discovery^13^. Neuroprotectants can modulate cell survival or death pathways common to many iRD, offering potential widespread applicability, irrespective of the genetic cause.

Previously reported retinal neuroprotectants include rod cone viability factor (rdCVF)^14^, brain-derived neurotrophic factor (BDNF), ciliary neurotrophic factor (CNTF)^15^, nicotinic acetylcholine or serotonin receptor agonists^16, 17^ and histone deacetylase inhibitors (HDACi). HDACi are clinically administered as mood stabilisers or anti-epileptic agents^18^ and are in clinical trials as anti-cancer^19^ or anti-inflammatory^20^ drugs. Notably, HDACi are under investigation to treat neurodegeneration in Alzheimer’s and Huntington’s disease^21^. Not suprisingly, HDACi recently emerged as potential drugs for iRD. A retrospective review of retinitis pigmentosa patients prescribed the HDACi valproic acid (VPA), showed improved visual field (VF) and visual acuity (VA) over an average 4 month treatment period^22^. The average optotype increased from 20/47 to 20/32. The study noted no negative effects on either VF or blood chemistry. Improvements in VF were clinically significant and indicates that VPA prevents progressive peripheral-central degeneration of retinal photoreceptor cells. However, multiple study criticisms include the small sample size, the short study period and the statistical methodology^23^. A similar retrospective review of a 9.8 month average treatment study suggests VPA has no beneficial effect on VF or VA readings^24^. Sisk *et al*. suggest HDACi treatment is appropriate for autosomal dominant RP acting to chaperone mutant rhodopsin, but may be harmful in autosomal recessive RP^25^. In summary, there is growing but conflicting evidence that HDACi are neuroprotective in a wide range of pre-clinical models of RP, retinal ischemia and retinal drug induced excitotoxicity^26–28^.

Zebrafish provide novel opportunities to efficiently investigate vertebrate vision and pharmacological interventions *in vivo* ^29^. At 3 days post fertilisation (dpf), ocular morphogenesis is largely complete and a differentiated laminated retina prevails^30^. Vision in zebrafish larvae is cone-mediated, unlike nocturnal rodent models whose vision is largely rod-mediated^31^. The zebrafish retina is abundant in cone photoreceptors, arranged in mosaic patterns of red/green, blue, and UV subtypes in the photoreceptor cell layer^32^ and zebrafish models of inherited blindness (e.g. *zatoichi* ^*s125*^, *pde6c* ^*w59*^ and *no optokinetic response f* ^*w21*^) demonstrate that genes essential for cone function (*gc3*, *pde6* and *gnat2*) are functionally conserved between fish and humans^33–35^. Convenient methods to assess visual function in zebrafish include behavioural assays or direct retinal electrophysiology^36–38^.

Here, we demonstrate pharmacological rescue of cone-mediated vision in the *dye* ^*ucd6*^ (https://zfin.org/ZDB-ALT-170502-2) zebrafish model of inherited blindness. HDACi treatment results in a rescue of some gross and retina-specific morphological defects, and significant improvement in visual function. Interrogation of proteomic datasets from treated and control *dye* mutants identified a correlation between upregulation of BDNF-TrkB signaling and HDACi mediated rescue of vision. The functional significance of BDNF-TrkB signaling was validated by reversal of HDACi-mediated improved vision when co-treated with ANA-12, a pharmacological antagonist of the TrkB receptor. Most significantly, treatment with a BDNF mimetic, 7,8-dihydroxyflavone hydrate (7,8-DHF), was sufficient to rescue visual function in *dye* larvae. In summary, characterisation of the mechanisms by which histone deacetylase inhibitors (HDACi) rescue visual impairment uncovered BDNF-TrkB signalling as necessary and sufficient to restore visual function in a model of inherited blindness.

## Results

### *dye* mutants display aberrant retinal morphology

**Figure 1:**
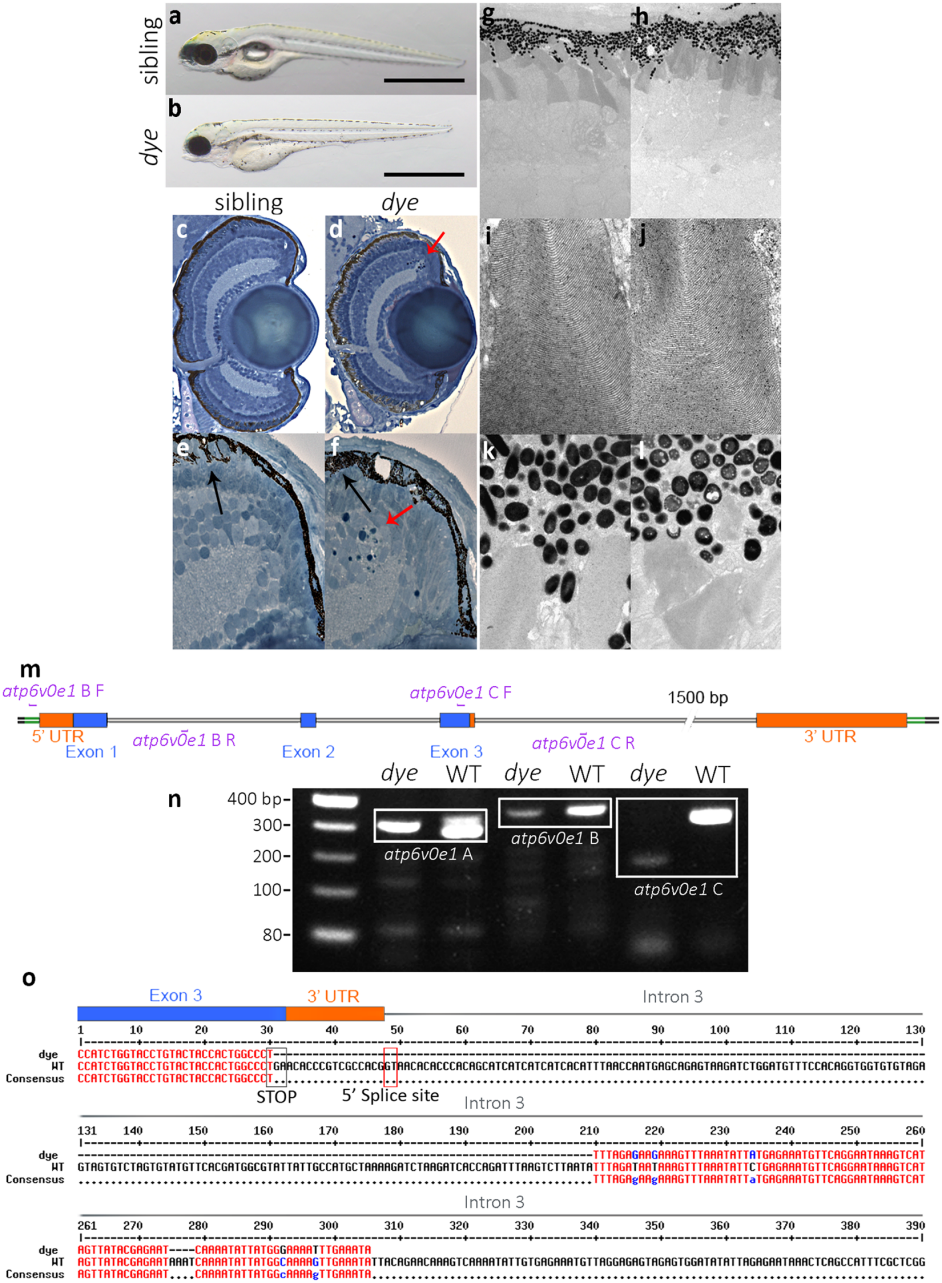
*dye* mutants display aberrant retinal morphology. Representative lateral and dorsal views in brightfield of sibling larvae (**a,a′**) and *dye* mutants (**b,b′**), displaying pigmentation and gross morphological defects present in the *dye* mutant, including swim bladder deflation compared to sibling (*in **a**) and pericardial oedema (black arrow in **b**). Transverse sections of the retina and ciliary marginal zone of sibling larvae (**c,e**) and *dye* mutants (**d,f**), pyknotic nuclei (red arrows in **d,f**) present in the ciliary marginal zone indicated dying cells. Transmission electron micrographs of photoreceptors in sibling larvae (**g**) and *dye* mutants (**h**) displaying shortened photoreceptor OS and aberrant morphology despite preserved ultrastructure (**i,j**). RPE cells in *dye* mutants are hypopigmented and contain large inclusion bodies compared to sibling larvae (**k,l**), and the RPE fails to interdigitate with photoreceptors in *dye* compared to siblings (N = 1 for sibling, N = 3 for *dye* mutants). (**m–o**) The mutation in *dye* affects the zebrafish *atp6v0e1* gene. (**m**) Schematic representation of the exon-intron structure of *atp6v0e1*, purple lines indicate the annealing position of genotyping primers used in panel **n**. (**n**) 1% agarose gel of PCR products from different regions of the *atp6v0e1* gene, a 180 bp deletion (white box, **n**) is detected in the region containing the terminal end of coding exon 3, the full length agarose gel image is included in the supplementary information (Supplementary Fig. S3a). (**o**) Sequencing of gel extracted PCR products and pairwise sequence alignment to the reference genome sequence (assembly GRCz11) revealed that the deletion removes the stop codon in, and the 3′ UTR encoded in exon 3 and of the GT splice donor site encoded at the exon 3-intron 3 border.

The *dying on edge* (*dye*) mutant was uncovered during an F_3_ N-ethyl-N-nitrosourea (ENU) mutagenesis screen in zebrafish. At 5 days post fertilization (dpf), *dye* larvae exhibit slightly decreased body length and eye diameter, hypopigmentation, pericardial oedema and deflated swim bladder (Fig. 1a,b). In ultrathin retinal sections, *dye* display increased cell death in the CMZ indicated by pyknotic nuclei (Fig. 1d,f). In comparison to wildtype siblings (Fig. 1c,e), *dye* photoreceptor outer segments (OS) are decreased in length, lack an orderly tiered layering and are not orientated perpendicular to the retinal pigment epithelium (RPE) (Fig. 1g,h). However, the OS ultrastructure presents with normal stacking of outer segment membranes (Fig. 1i,j). The RPE of *dye* is hypopigmented, and forms large intracellular inclusion bodies (Fig. 1k,l). Positional cloning localised *dye* to the *atp6v0e1* gene and the causative mutation was identified as a 180 bp deletion (Fig. 1m–o) that removes the stop codon, a 15 bp sequence of the 3′ UTR, and the donor splice site from the exon 3-intron 3 boundary that is required for correct splicing of the *atp6v0e1* mRNA (Fig. 1o). In agreement, RT-PCR demonstrated a large reduction of *atp6v0e1* mRNA expression in *dye* compared to wildtype larvae (Supplementary Fig. S1) and similar morphological defects are present in other vacuolar ATPase subunit mutants^39^.

### *dye* mutants have significantly impaired cone photoreceptor-mediated visual function

**Figure 2:**
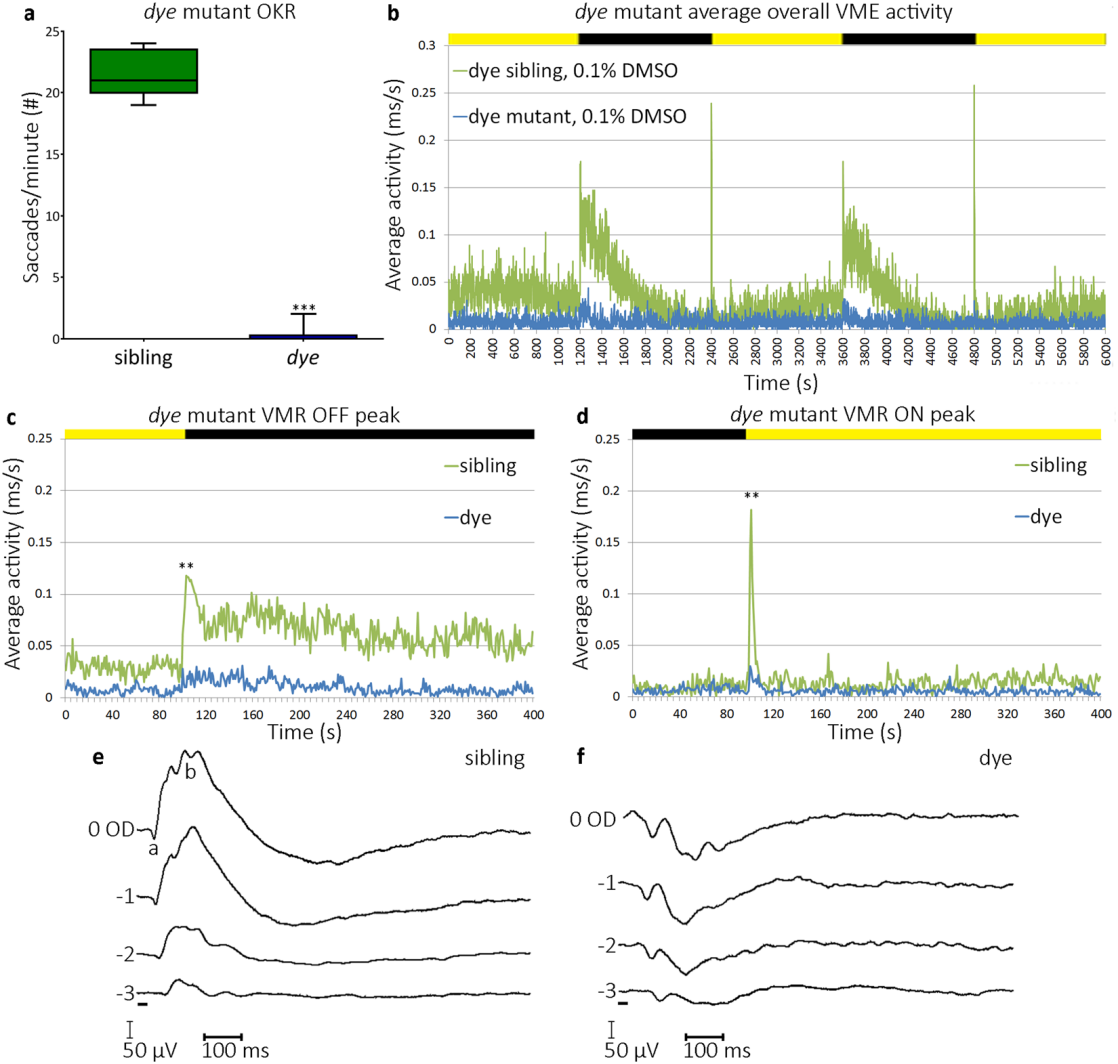
*dye* mutants exhibit reduced/absent visual behaviour. (**a**) *dye* mutants have a reduced/absent OKR compared to unaffected siblings, data represented using a box and whiskers plot, N = 15 (number of larvae per replicate), n = 3 (number of replicates). (**b–d**) Average overall VMR activity of *dye* mutants (blue trace) and siblings (green trace), through the 1 h 40 minute experiment (**b**), graph depicts average activities of 12 larvae in 3 separate replicates. Peak activity in response to a transition from light to dark (**c**), and dark to light (**d**). Graphs in **c** and **d** depict the average activity from 2 transitions in one experiment and the average across 3 separate replicates, yellow and black bars represent dark and light periods. N = 12, n = 3 for VMR assays. Electroretinograms (**e,f**) depict sibling and *d* *ye* responses to 20 ms flashes (black bar) at increasing light intensities (−3.0, −2.0, −1.0 and 0 optical densities), with a maximum light intensity of 2.8 × 10^3^ µW/cm^2^. Statistical analysis was carried out using a student’s t-test with unequal variances, ***p < 0.001, **p < 0.01.

To determine if *dye* have reduced visual function, we conducted OKR and VMR assays (Fig. 2). The OKR measures the ability to track objects by quantifying the number of saccadian eye movements a larva exhibits in response to a rotating grated stimulus^36^. Notably, 5 dpf *dye* display a ~98% reduction in OKR, producing an average of 0.33 (±0.67) saccades per minute (sacc./min.) compared to an average of 21.6 (±1.6) sacc./min. in unaffected siblings^+/−,+/+^ (Fig. 2a). The VMR quantifies larval locomotor activity in response to sudden changes in lighting^37^. Rapidly changing the environment to illuminated (ON response) or darkened (OFF response), results in a ‘startled’ behavioural response wherein locomotor activity is acutely increased. 5 dpf *dye* (blue trace, Fig. 2b) show reduced VMR activity with significantly reduced ON and OFF peak responses and no elevated light seeking behaviour in dark conditions compared to siblings (green trace, Fig. 2b). *d* *y* *e* exhibit a 79% reduction in the average MAX OFF VMR peak producing an average activity response of 0.055 (±0.013) ms/s compared to 0.259 (±0.049) ms/s in unaffected siblings (Fig. 2c). *dye* display an 85% reduction in the MAX ON VMR peak, responding with an average activity of 0.054 (±0.011) ms/s compared to 0.354 (±0.068) ms/s in unaffected siblings (Fig. 2d). *dye* also exhibit a defective electroretinogram (ERG), a more direct measurement of outer retinal function (Fig. 2e,f), with *dye* displaying reduced or absent b-waves in response to 20 millisecond flashes of increasing light intensity. In summary, visual behaviour assays and ERG measurements confirm *dye* have significantly impaired visual function. Notably, initiation of visual behaviour and retinal function in zebrafish larvae is predominantly mediated by cone and not rod photoreceptors^35^.

### HDACi treatment restores retinal morphology and visual function in *dye*

**Figure 3:**
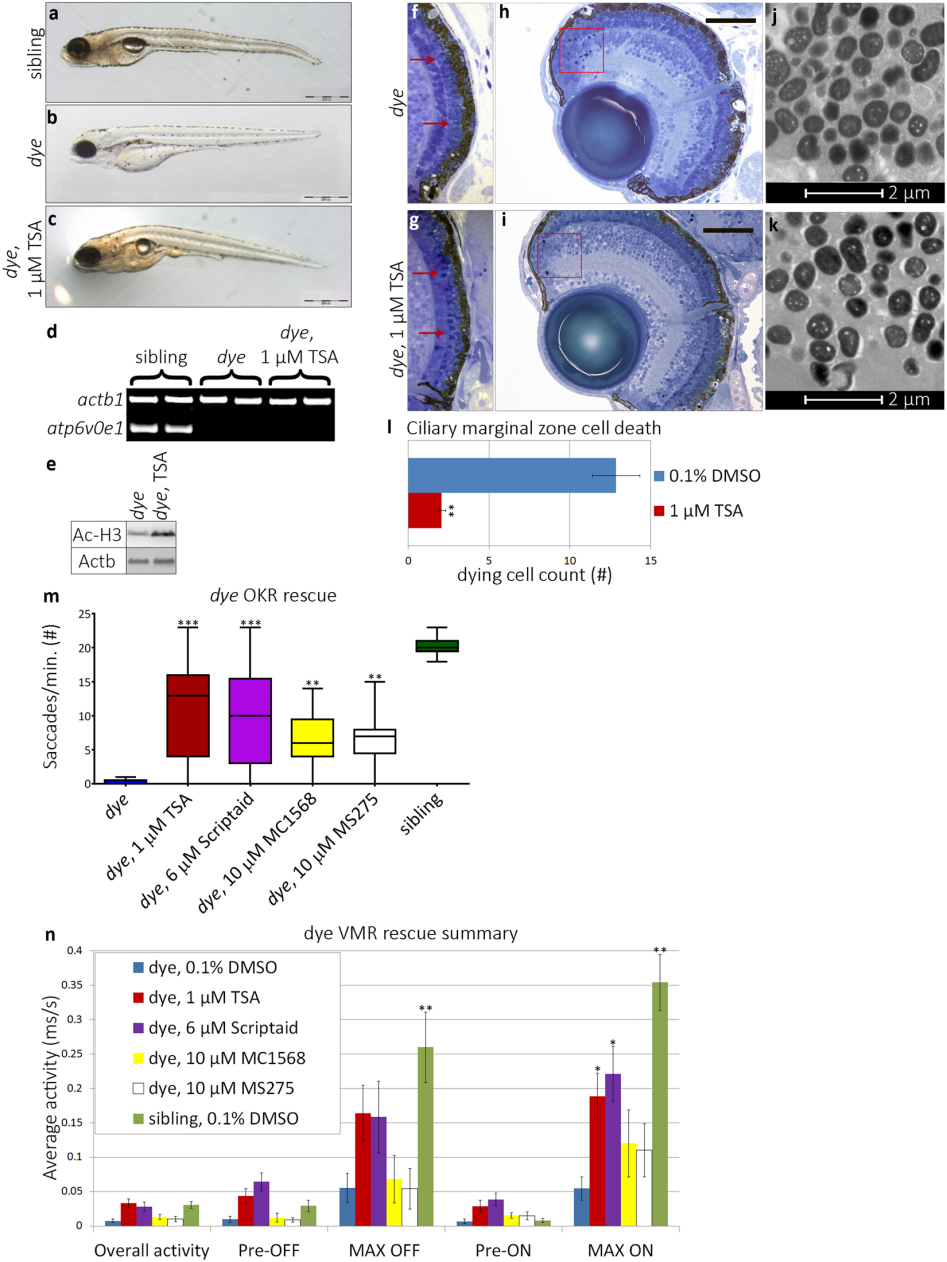
HDACi treatment rescues morphological and visual behaviour defects in *dye* mutants. (**a**–**c**) Lateral bright field images of sibling control (**a**), *dye* mutant control (**b**) and 1 µM TSA treated *dye* (**c**). TSA treatment partially rescued gross morphological defects, restoring pigmentation, eye size and swim bladder inflation. Scale bars represent 500 µm scale. (**d**) The *dye* genotype was confirmed by the absence of a PCR product from the *atp6v0e1* gene, *B-actin* served as a positive control. (**e**) Increased histone H3 acetylation was observed by western blot in TSA treated *dye* larvae compared to control. (**f,g**) Light micrographs of *dye* retinal sections. Photoreceptor outer segments are less visible in control treated *dye* (**f**) compared to 1 µM TSA treated larvae **(g)** (red arrows, **f,h**). (**h,i**) Cell death in the ciliary marginal zone is reduced in 1 µM TSA treated *dye* mutants (**i**), indicated by a reduction in the number of pyknotic nuclei present (red boxes) compared to *dye* mutant control (**h**), scale bars represent 100 μm scale. (**j,k**) Electron micrographs of ultra-thin (500 nm) *dye* retinal sections, 1 µM TSA treatment (**k**) does not significantly rescue RPE hypopigmentation or reduce the number of RPE cells that contain inclusion bodies in comparison to *dye* mutant controls (**j**). (**l**) The number of pyknotic nuclei through multiple sections from the central retina and individual larvae is significantly reduced, N = 5, n = 3. (**j**) HDACi treated *dye* mutants have significantly improved OKR following treatment, N = 12 (number of larvae per replicate), n = 3 (number of replicates). (**k**) Summary of VMR activities, 1 µM TSA and 6 µM Scriptaid significantly improve the MAX ON activity, N = 12, n = 3. Statistical analyses were performed using a Kruskal-Wallis one-way analysis of variance and post-hoc Dunn’s multiple comparison test, comparing each group to 0.1% DMSO treated *dye* mutants, error bars represent average SEM, ***p < 0.001, **p < 0.01, *p < 0.05. Full length agarose gels and western blots are included in the supplementary information (Supplementary Fig. S3b–d).

As previous studies report histone deacetylase inhibitors (HDACi) to preserve retinal morphology in mouse models of rod photoreceptor degeneration^26^, we investigated the effects of a panel of HDACi on retinal morphology and cone-related visual function in *dye*. Initial dose-range finding (0.25–4 µM) assays indicated that 1 µM trichostatin A (TSA) was the maximum tolerated dose for zebrafish larvae submersed in the drug diluted in embryo medium. Strikingly, treatment with TSA acutely reversed many morphological defects observed in *dye* (Fig. 3a–i). Homozygous *dye* larvae treated with 1 µM TSA from 3–5 dpf present with increased tissue pigmentation, eye size and body length; inflated swim bladders; and reduced pericardial oedema (Fig. 3a–c). The genotype of *dye* larvae was validated by PCR showing that homozygous *dye* mutant DNA, but not sibling DNA, was negative for a 528 bp PCR amplicon encompassing exon 3 of the *atp6v0e1* gene. By contrast, a β-actin control amplicon was present in all samples (Fig. 3d). Treatment with TSA also increases acetylation of histone 3 in *dye* (Fig. 3e). In the retina, the number of prominent photoreceptor outer segments is markedly increased following HDACi treatment (Fig. 3f,g). Cell death in the CMZ and peripheral retina is significantly reduced as evidenced by a reduction in the number of pyknotic nuclei (Red boxes, Fig. 3h,i, quantified in l) present in serial transverse sections from the central retina. However, RPE hypopigmentation and the presence of inclusion bodies is not rescued (Fig. 3j,k).

To determine if HDACi treatment can restore cone photoreceptor-mediated visual function in *dye*, visual behaviour in response to treatment with different HDACi was quantified using OKR and VMR assays (Fig. 3m,n). The average OKR response of *dye* is significantly rescued in response to 1 µM TSA (10.69 ± 2.12 sacc./min.) a HDAC class I/II inhibitor, 6 µM Scriptaid (9.53 ± 1.96 sacc./min.) a HDAC pan-inhibitor, 10 µM MC1568 (6.4 ± 2.66 sacc./min.) a HDAC class II selective inhibitor or 10 µM MS-275 (6.51 ± 2.24 sacc./min.) a HDAC class I selective inhibitor representing 42.8, 38.1, 25.2 and 25.7 fold average increases, respectively (Fig. 3m). In VMR assays, the MAX ON response is significantly rescued with 1 µM TSA (0.189 ± 0.058 ms/s) or 6 µM Scriptaid (0.221 ± 0.071) representing 2.5 and 3.1 fold increases in VMR, respectively (Fig. 3n). MC1568 and MS-275 also increase the MAX ON response but this did not achieve statistical significance. In summary, HDACi, particularly Class I/II inhibitors, rescue retinal morphology and restore visual behaviour in the *dye* model of inherited blindness.

### Proteomic profiling, the HDACi molecular mechanism of rescue

**Figure 4:**
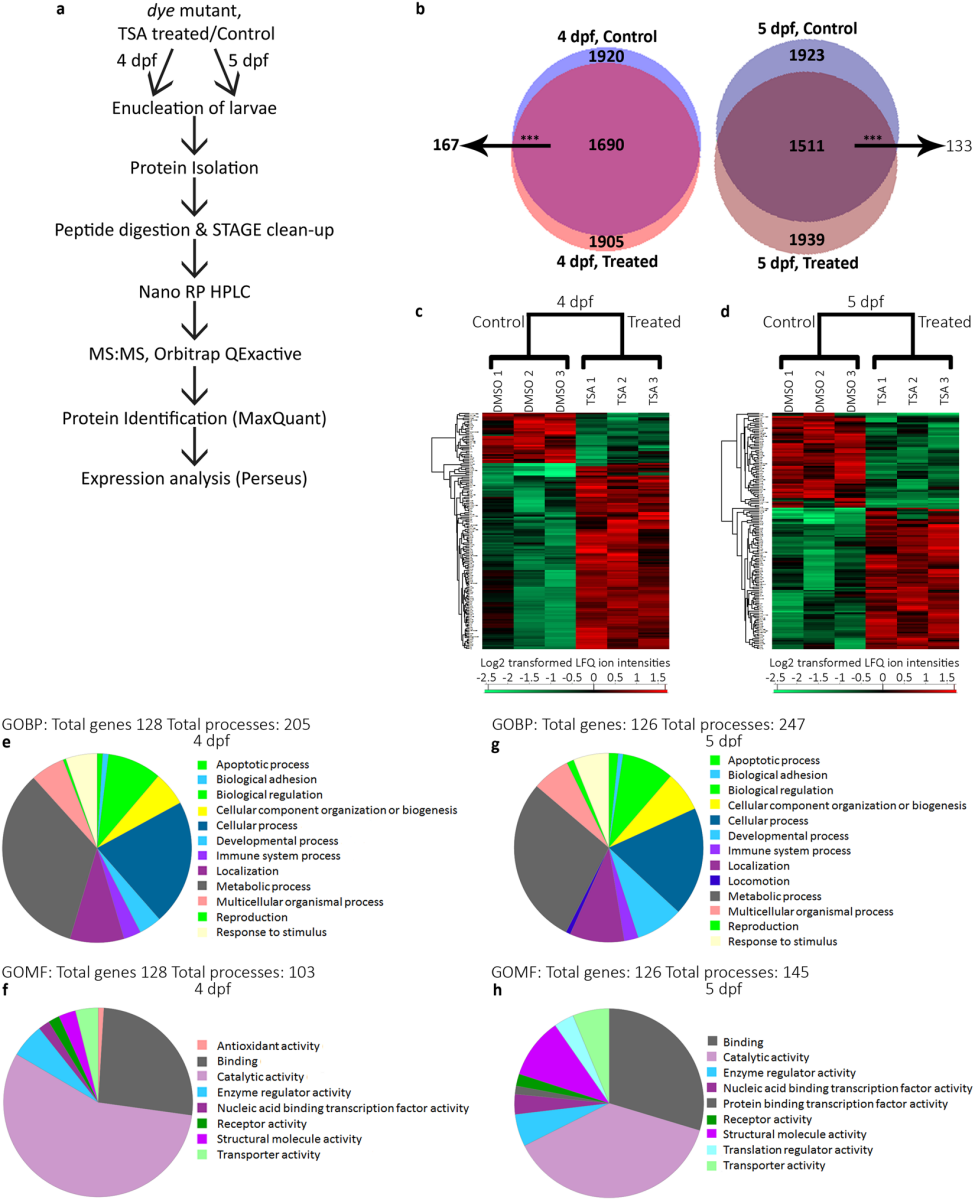
Identification of differentially expressed proteins in the eye following TSA treatment in *dye* mutants. (**a**) Schematic representation of LC:MS/MS experiments and analysis. 25 larvae from *dye* control and TSA treated groups were enucleated at 4 and 5 dpf treatment end points, proteins were extracted and prepared for analysis by LC:MS/MS, experiments were performed with three biological replicates for each group. (**b**) Venn diagram of total proteins identified. 1920 proteins were identified in the 4 dpf control group and 1905 proteins identified in the 4 dpf TSA treated group. 1690 proteins overlapped between groups at this time point of which 147 were significantly differentially expressed. 1923 proteins were identified in the 5 dpf control group and 1939 proteins identified in 5 dpf TSA treated. 1511 proteins overlapped between groups at this time point of which 131 were significantly differentially expressed (**c,d**) p < 0.05. Colour-scale heat maps of the significantly differentially expressed proteins identified in at least 2 biological replicates at both treatment time points based on log_2_ transformed LFQ values. At 4 dpf, 32 proteins are decreased and 115 proteins are increased following TSA treatment (**c**). At 5 dpf, 51 proteins are decreased and 80 increased following TSA treatment (**d**), red colour indicates higher abundance, green indicates lower abundance. Comparisons were made by ANOVA with a p-value cut off of p < 0.05. (**e–h**) Pie charts displaying gene ontology (Biological process [top] and Molecular Function [bottom]) terms for significantly differentially expressed proteins from T1 (4 dpf, **e,f**) and T2 (5 dpf, **g,h**) generated from PANTHER-DB.

To mechanistically understand how HDACi mediate rescue of visual function in *dye*, protein expression in the eye was profiled by mass spectrometry. 3 dpf *dye* larvae were treated with 1 µM TSA or vehicle control (0.1% DMSO) until 4 or 5 dpf. At 4 dpf, 1920 and 1905 proteins were identified from eyes of vehicle control or 1 µM TSA-treated *dye*, respectively (Fig. 4a, Supplementary data S1). Of the 1690 overlapping proteins within these datasets, 147 show significant (Fisher exact test p < 0.05) differential expression; 32 were decreased and 115 increased (Fig. 4b, Table 1). At the 5 dpf end point, 1923 and 1939 total proteins were identified from eyes of vehicle control or 1 µM TSA treated groups, respectively (Fig. 4a, Supplementary data S1). Of the 1511 overlapping proteins identified in both test groups, 131 proteins are significantly (Fisher exact test p < 0.05) differentially expressed; 51 decreased and 80 increased (Fig. 4c, Table 2). Cluster analysis of the differentially expressed proteins highlighted the concordance of the triplicate experiments for vehicle- or TSA-treated samples and identified sub-groups of ocular proteins exhibiting equivalent alterations in protein expression post HDACi treatment (Fig. 4b). To determine biological processes contributing to HDACi mediated rescue, enriched gene ontology terms were identified in DAVID-DB (Supplementary data S2) and PANTHER-DB (Fig. 4d,e) databases. For the significantly differentially expressed proteins at both time points, processes involved in initiation of transcription (nucleotide binding), protein translation (aminoacyl-tRNA ligase activity), visual perception and metabolism are over-represented in the dataset.

**Table 1:** Significantly differentially expressed eye proteins in TSA treated *dye* at 4 dpf.

4 dpf fold-change (1 µM TSA vs. 0.1% DMSO)	ANOVA p- value	UniProt ID	Protein Names	Gene name
1.42	9.80E-04	B0UYN4	Zgc:162944 protein	*zgc:162944*
−1.17	1.72E-03	Q6NSP0	Thyroid hormone receptor associated protein 3b	*thrap3b*
1.51	1.96E-03	Q6NYQ7	Nicotinamide nucleotide transhydrogenase protein (Fragment)	*nnt*
2.10	2.07E-03	Q6PFN9	Glycine dehydrogenase (decarboxylating)	*gldc*
1.37	2.59E-03	Q566N7	Sulfide quinone reductase like (Yeast)	*sqrdl*
2.39	2.80E-03	F1QSL9	Guanylate cyclase	*gc3*
1.77	2.98E-03	Q1XHM0	G protein-coupled receptor kinase 1 a	*grk1a*
−1.60	3.09E-03	Q6DRQ1	Si:ch211-114c12.2	*si:ch211-114c12.2*
1.37	3.15E-03	B3DLK1	exportin 7	*xpo7*
1.48	3.27E-03	E7F1R1	Pyruvate carboxylase	*pcl*
2.01	3.51E-03	Q52JI6	Beta A1-2-crystallin	*cryba1l*
1.44	3.93E-03	Q2LEK1	Dynein cytoplasmic 1 heavy chain 1	*dync1h1*
1.67	3.96E-03	A2CER9	Ankyrin repeat and FYVE domain containing 1	*ankfy1*
−3.46	5.17E-03	F1REH0	Vinculin	*vcl*
1.29	5.20E-03	A9ULS7	Solute carrier family 2 (facilitated glucose transporter), member 11b	*slc2a11b*

**Table 2:** Significantly differentially expressed eye proteins in TSA treated *dye* at 5 dpf.

5 dpf fold-change (1 µM TSA vs. 0.1% DMSO)	ANOVA p-value	UniProt ID	Protein Names	Gene name
2.50	2.26E-05	A9JRA5	Scinderin like a	*scinla*
−1.76	2.77E-04	F1RA50	Cathepsin D	*ctsd*
1.39	3.95E-04	F1QX42	Cleavage and polyadenylation specific factor 2	*cpsf2*
2.85	4.97E-04	F1QZW1	Ventricular myosin heavy chain	*vmhc*
−2.45	8.29E-04	Q9DDJ2	Caspase (Fragment)	*caspb*
−1.35	1.20E-03	F1QWA4	Influenza virus NS1A-binding protein homolog B	*ivns1abpb*
−1.63	1.33E-03	Q5R2V3	Gamma-D-crystallin (Fragment)	*crygn2*
−1.43	2.06E-03	I3IRW7	4-Aminobutyrate Aminotransferase	*abat*
−1.64	2.14E-03	Q1LUU8	Zinc finger protein 384 like	*znf384l*
1.58	2.76E-03	F1QRG5	Uncharacterized protein	*si:dkey-250d21.1*
1.69	2.79E-03	Q1LV08	Nucleoporin 93 kDa	*nup93*
1.62	3.30E-03	F1R9Y8	High density lipoprotein-binding protein (Vigilin)	*hdlbp*
1.60	3.36E-03	Q499A8	Poly (ADP-Ribose) Polymerase 1	*parp1*
1.38	3.46E-03	E7F8S4	Transketolase-like	*LOC557518*
1.36	4.60E-03	E7FD56	N(alpha)-acetyltransferase 25, NatB auxiliary subunit	*naa25*
1.33	4.76E-03	Q6PFM3	Solute carrier family 25 (aspartate/glutamate carrier), member 12	*slc25a12*

Differentially expressed proteins in response to HDACi treatment are linked to diverse functions including: *i*) anti-oxidant activity (mitochondrial NAD(P) transhydrogenase (Nnt): 1.5 fold increase); *ii*) metabolism (pyruvate carboxylase (Pcl): 1.48 fold increase); *iii*) mRNA processing (cleavage and polyadenylation specific factor 2 (Cpsf2): 1.4 fold increase), *iv*) transport (exportin 7 (Xpo7): 1.4 fold increase and solute carrier family members (Slc2a11b and Slc25a12): 1.3 fold increases), and *v)* unknown functions (Si:ch211-114c12.2: 1.6 fold decrease and Zgc:162944 protein: 1.4 fold increase).

Consistent with the improvement of visual function, many proteins increased in *dye* eyes following HDACi treatment are key mediators of phototransduction. These include: (a) light-sensitive G-protein coupled receptors, rhodopsin and opsin 1 short/medium/long wave sensitive 1/2 (Rho, Opn1sw1, Opn1mw2, Opn1lw1) exhibiting 1.8, 1.4, 1.7 and 1.6 fold increases, respectively); (b) visual effector G proteins, guanine nucleotide-binding protein G(t) subunit alpha-1/2 (Gnat1/2) exhibiting 1.5 and 1.2 fold increases, respectively); (c) phosphodiesterase a/b/c isoforms (Pde6a/b/c) exhibiting 1.6, 2.5 and 1.7 fold increases, respectively); (d) guanylyl cyclase 3 (Gc3) exhibiting a 2.4 fold increase); and (e) G protein-coupled receptor kinase 1 (Grk1) exhibiting a 1.8 fold increase). Other HDACi modulated proteins control photoreceptor development and morphogenesis including: peripherin 2 a/b (Prph2a/b) with 1.6 and 1.5 fold increases; dynein cytoplasmic 1 heavy chain 1 (Dync1h1) with 1.4 fold increase; lens crystallin beta A1-A2 Crystallin, scinderin like a, and gamma D-crystallin (Cryba1l, Scinla, Crygn2) with 2 and 2.5 fold increase or 1.6 fold decrease, respectively). Significantly differentially expressed proteins linked with neuroprotection include: heat shock protein 90 (Hsp90) with a 1.7 fold decrease and heat shock protein 70 (Hsp70) with a 1.3 fold increase. There is also a reduction in Caspase 3 (1.7 fold decrease) that correlates with the reduced cell death observed in the CMZ of *dye* mutants in response to HDACi treatment.

### BDNF-TrkB signaling is central to HDACi mediated rescue in the *dye*

**Figure 5:**
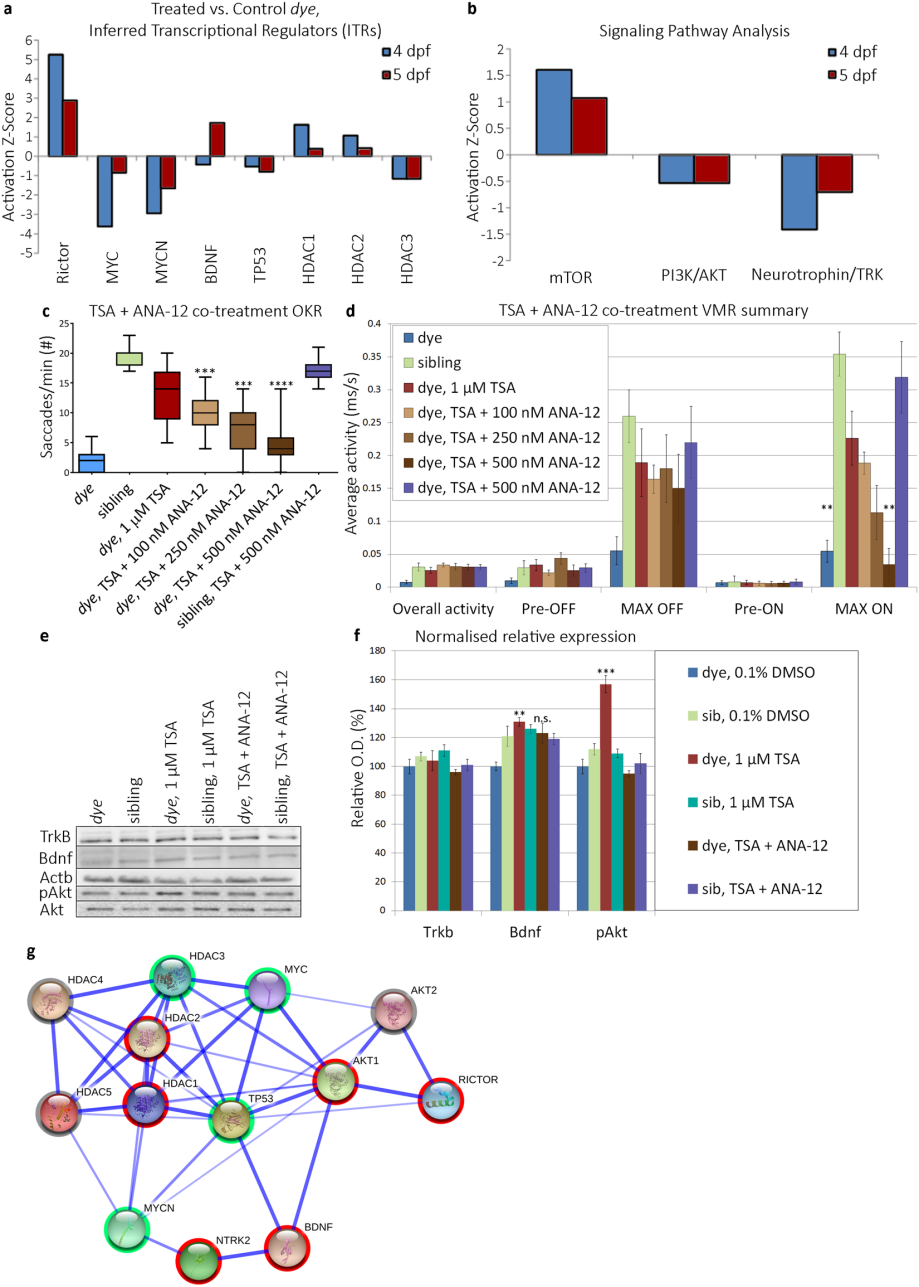
Identification and validation of HDACi mediated rescue pathway. (**a**) Activation/inhibition z-score plot of inferred transcriptional regulators (ITRs) in response to TSA treatment, at 4 and 5 dpf treatment end points in the *dye* mutant. ITRs were inferred by IPA from the HDAC inhibitor LC/MS:MS data. (**b**) Activation/inhibition z-score plot of identified HDACi modulated pathways, activation status is determined by abundance of downstream effector proteins present in the LC/MS:MS dataset. (**c,d**) Visual behaviour assay responses following co-treatment of TSA and ANA-12, an antagonist of the TrkB receptor, ANA-12 co-treatment results in a dose-dependent reduction in OKR (**c**). (**d**) Summary of VMR activity, ANA-12 co-treatment results in a dose-dependent reduction in the MAX ON response, changes in visual behaviour are compared to TSA only treated *dye* larvae, statistical analysis was performed using a Kruskal-Wallis one-way analysis of variance and post-hoc Dunn’s multiple comparison test, comparing each group to TSA only treated *dye* mutants. (**e,f**) Validation of differential expression of Bdnf, TrkB and effectors in the signaling pathway in 25 pooled eyes by western blot. TSA treatment increased expression of Bdnf, but not Trkb and this expression pattern was not affected by ANA-12 co-treatment. Akt (Thr308) phosphorylation was increased in TSA treated *dye*, while levels remained unchanged in ANA-12 co-treated larvae, N = 25 (number of eyes per replicate), n = 3 (number of replicates). Images represent a typical blot, bar chart represents signal intensity measured by densitometry, Trkb and Bdnf signals normalised to Actb expression and pAKT (Thr308) signal normalised to total Akt. Statistical analysis was performed using a two-tailed unpaired t-test in comparison to *dye* control. Error bars indicate S.E.M, ****p < 0.0001 ***p < 0.001, **p < 0.01. Full length western blots are included in supplementary information (Supplementary Fig. S3e–i). (**g**) Protein interaction map (generated by STRING-DB) of ITRs differentially expressed upon TSA treatment. Akt1, Akt2 and TrkB proteins were added to the network based on ITR pathway analysis and western blot data. Activated nodes are highlighted with red, downregulated nodes with green, grey highlighted nodes are predicted regulators but in which the direction is unknown.

In order to delineate the signaling pathways mediating HDACi rescue of *dye*, the proteomic datasets were analysed using Ingenuity Pathway Analysis (IPA) software. Upstream regulators of the effects observed in the dataset are inferred from changes in expression in downstream proteins. Additionally, the activation state of these inferred transcriptional regulators (ITRs) was predicted based on the abundance of downstream effector proteins. Brain derived neurotrophic factor (BDNF), N-myc proto-oncogene (MYCN), myc proto-oncogene (MYC), tumor protein 53 (TP53) and RPTOR independent companion of MTOR, complex 2 (RICTOR) were identified as ITRs, as were several HDAC isoforms (Fig. 5a). In HDACi rescued *dye*, MYC, MYCN and TP53 signaling was predicted to be inhibited; in contrast, RICTOR and BDNF signaling was predicted to be activated (Fig. 5a). Consistent with this, BDNF-NTRK, PI3K and mTOR signaling were all differentially activated by our HDACi treatment (Fig. 5b). HDAC isoforms, HDAC1 and HDAC2 were predicted to be activated at the 4 dpf treatment endpoint, though this activation was reduced somewhat at the 5 dpf treatment endpoint. Conversely, HDAC3 is inhibited at both timepoints. As *bdnf* transcription is rapidly induced by HDACi treatment in cultured rat cortical neurons^40^ and is upstream of PI3K/AKT/RICTOR signaling we assessed the contribution of BDNF-TrkB signaling to in the rescue of vision in *dye*.

**Figure 6:**
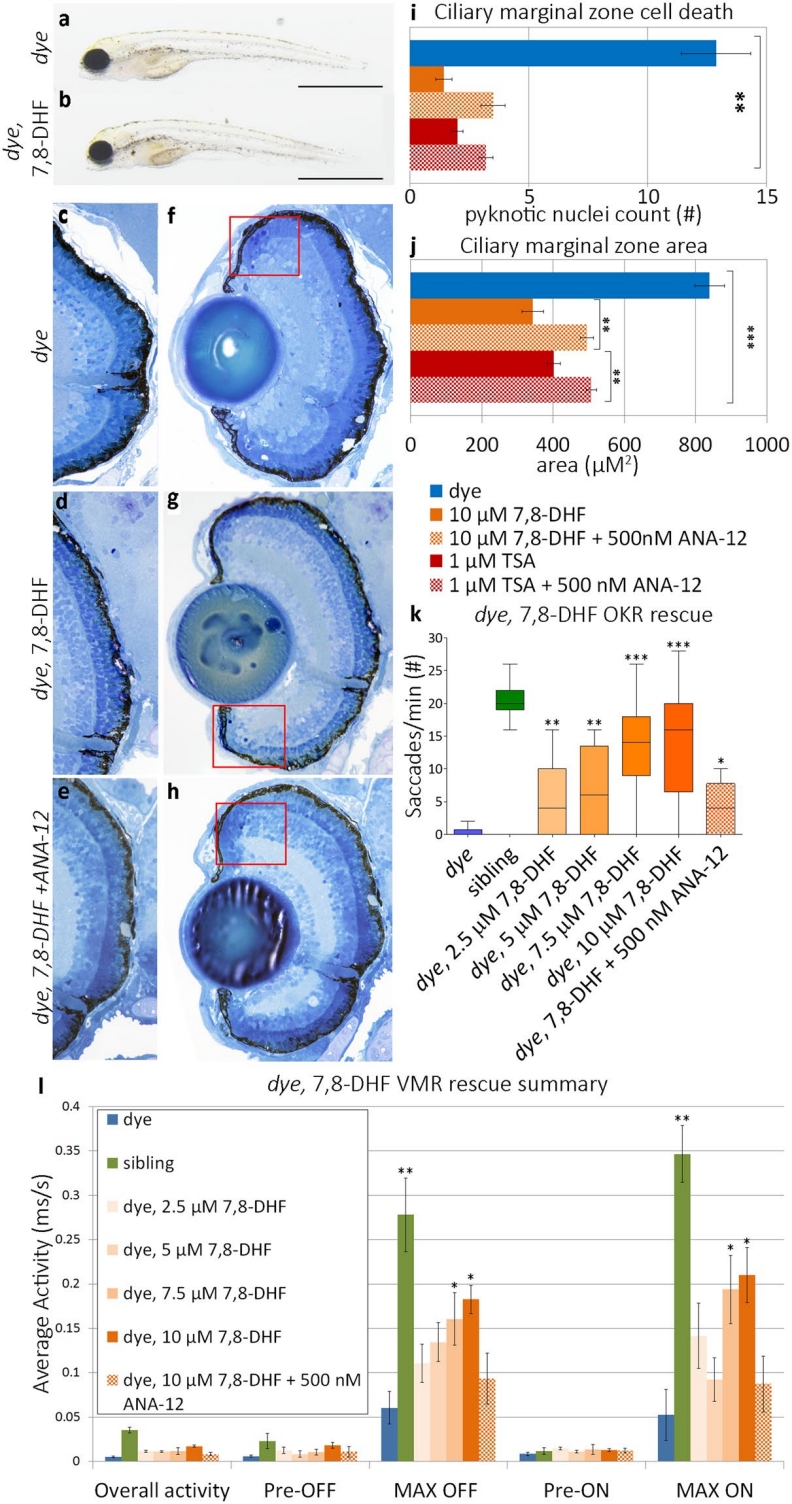
A BDNF mimetic treatment rescues visual function in *dye* mutants. (**a,b**) Gross morphology of control and 7,8-dihydroxyflavone hydrate (7,8-DHF) treated *dye* larvae, treatment does not rescue gross morphological defects. (**c–h**) Light micrographs of control, 7,8-DHF treated and 7,8-DHF + ANA-12 co-treated *dye* retinal sections. The number of pyknotic nuclei present in the ciliary marginal zone is significantly reduced following treatment with 7,8-DHF (rectangles **f,g**). This reduction in pyknotic nuclei is partially reversed by 7,8-DHF + ANA-12 co-treatment (**h**). (**i**,**j**) bar plots quantifying the average number of pyknotic nuclei present in multiple sections and replicate larvae following treatment (**i**) (N = 5, n = 3) and changes in area of the ciliary marginal zone in the central retina (n = 3) (**j**). (**k**) 7,8-DHF treated *dye* mutants have a significantly improved OKR, the degree of rescue is dose-dependent. (**i**) Likewise, treatment with 7,8-DHF rescues the VMR MAX ON and MAX OFF responses, statistically significant rescue was observed at 7.5 and 10 µM 7,8-DHF concentrations, N = 12 (number of larvae per replicate), n = 3 (number of replicates) for all groups. Visual function rescue was abrogated by ANA-12 co-treatment. Visual behaviour data was analysed by a Kruskal-Wallis one-way analysis of variance and post-hoc Dunn’s multiple comparison test, comparing each group to 7,8-DHF treated *dye* mutants, error bars represent average SEM. Comparison of pyknotic nuclei counts and comparison of CMZ area was performed using an ordinary one-way ANOVA, ***p < 0.001, **p < 0.01, *p < 0.05.

To validate the requirement of BDNF-TrkB signaling for the rescue of visual function, co-treatments were performed with ANA-12, a known pharmacological antagonist of the TrkB receptor. Visual response rescue following co-treatment with 1 µM TSA plus 100–500 nM ANA-12 was significantly reduced but not to untreated mutant levels, in a dose-dependent manner for both the OKR (69% maximum reduction) and MAX-ON response of the VMR (85% maximum reduction) compared to 1 µM TSA alone (Fig. 5c,d). ANA-12 co-treatment partially reversed the TSA-mediated rescue of cell death in the CMZ (Fig. 6i,j). To further validate the requirement of BDNF-TrkB, changes in protein expression in treated eye homogenates were examined (Fig. 5e,f **)**. In *dye* larvae treated only with 1 µM TSA, a significant 31% increase in Bdnf expression is observed compared to vehicle controls (Fig. 5e,f). In contrast, Bdnf expression was not significantly increased in 1 µM TSA + 500 nM ANA-12 co-treated *dye* larvae compared to vehicle controls (Fig. 5e,f). TrkB receptor expression remained consistent across treatments (Fig. 5e,f). As the PI3K/AKT pathway is activated downstream of BDNF-TrkB signaling, the phosphorylation status of Akt (Thr308) was examined. Compared to vehicle controls, 1 µM TSA treatment significantly increased phosphorylation of Akt by ~57%, which was blocked by 1 µM TSA + 500 nM ANA-12 co-treatment (Fig. 5e,f). Expression or phosphorylation of these proteins was not significantly altered in equivalent treatments of wildtype or heterozygous sibling larvae (Fig. 5e,f). A STRING-DB protein-protein interaction network was generated by combining the predicted or confirmed protein activation status or abundance in HDACi treated *dye* (Fig. 5g). Changes in expression/activation of proteins in this network are in agreement with activation of BDNF-TrkB signaling wherein HDACi treatment increases expression of BDNF, subsequently activating Ntrk2/TrkB then activation of Akt and inhibition of MycN, P53, Myc and HDAC3.

### A BDNF mimetic is sufficient to restore visual function in *dye*

Our data suggested HDACi mediated rescue was largely due to modulation of TrkB signaling via increased endogenous production of BDNF. Thus, *dye* were treated with a BDNF mimetic, 7,8-dihydroxyflavone hydrate (7,8-DHF). Treatment with 10 µM 7,8-DHF did not rescue gross morphological defects present in *dye* to a significant degree (Fig. 6a,b), conversely, retinal morphology was rescued (Fig. 6c,d,f,g) to a similar degree to HDACi treated *dye* larvae, including an 89% reduction in the number of dying cells present in the CMZ (red boxes, Fig. 6f,g, bar chart, i), and a 59% reduction in the size of the CMZ (j). Co-treatment with 500 nM ANA-12 partially blocked rescue of CMZ cell death (Fig. 6i, 72% reduction) and reduction in CMZ size (Fig. 6j, 41% reduction). Significantly, treatment with 10 µM 7,8-DHF rescued visual function as assessed by OKR (Fig. 6k) and VMR (Fig. 6l), in a dose-dependent manner. The degree of rescue with 7,8-DHF (57.58 fold increase in OKR, 3.89 and 3.33 fold increase in VMR MAX ON and MAX OFF activities, respectively) was greater than that observed by the most effective HDACi. In addition, co-treatment with 10 µM 7,8-DHF and 500 nM ANA-12 abrogated rescue of visual behaviour (71% reduction in OKR, 48% reduction in MAX OFF and 58% reduction in MAX ON VMR) (Fig. 6k,l). A comprehensive analysis of the visual behaviour responses, including dose-dependent improvements in OKR following HDACi or BDNF mimetic treatment is presented in Table 3.

**Table 3:** Summary of visual behaviour responses of HDACi treated *dye* mutants.

Treatment	*dye* OKR (sacc./min.)	*dye* Average MAX ON (ms/s)	*dye* Average MAX OFF (ms/s)
0.1% DMSO	0.24 ± 0.76	0.054 ± 0.029	0.055 ± 0.037
0.25 µM TSA	0.83 ± 0.92	—	—
0.5 µM TSA	7.75 ± 2.19	—	—
1 µM TSA	**10.69 ± 2.12**	**0.189 ± 0.058**	0.164 ± 0.069
2 µM TSA*	9.25 ± 4.78	—	—
4 µM TSA*	5.75 ± 4.48	—	—
2 µM Scriptaid	2.38 ± 1.19	—	—
4 µM Scriptaid	5.75 ± 1.67	—	—
6 µM Scriptaid	**9.53 ± 1.96**	**0.221 ± 0.071**	0.158 ± 0.090
8 µM Scriptaid	6.5 ± 3.21	—	—
10 µM Scriptaid*	4.33 ± 0.66	—	—
5 µM MC1568	2.25 ± 0.71	—	—
10 µM MC1568	**6.4 ± 2.66**	0.120 ± 0.084	0.068 ± 0.059
15 µM MC1568	4.75 ± 2.12	—	—
20 µM MC1568	4.00 ± 1.6	—	—
5 µM MS275	3.13 ± 1.46	—	—
10 µM MS275	**6.51 ± 2.24**	0.110 ± 0.069	0.054 ± 0.051
15 µM MS275	4.38 ± 2.07	—	—
20 µM MS275	3.88 ± 1.46	—	—
1 µM VPA	0.44 ± 0.66	—	—
2.5 µM 7,8-DHF	5.75 ± 1.12	0.142 ± 0.107	0.110 ± 0.055
5 µM 7,8-DHF	6.42 ± 1.23	0.092 ± 0.109	0.134 ± 0.072
7.5 µM 7,8-DHF	13.39 ± 0.67	0.194 ± 0.128	0.160 ± 0.075
10 µM 7,8-DHF	**14.06 ± 0.8**	**0.210 ± 0.085**	**0.183 ± 0.076**

*Toxic concentration, affecting survival & gross morphology. Bold text indicates p-value < 0.001.

In summary, pathway analysis of *dye* proteomic datasets suggested BDNF-TrkB signaling as a mediator of HDACi rescue of visual function. The requirement and sufficiency of BDNF-TrkB signaling for rescue of visual function was confirmed experimentally by pharmacological intervention, biochemical analysis and visual behaviour assays.

## Discussion

Currently, there are no approved pharmacological interventions to stabilise or improve visual function in patients with inherited blindness. Advantages of drug treatment include the ability to fine-tune effective and safe concentrations for each patient, to co-administer drug combinations, to incorporate sustained drug delivery devices and to stop treatment if ineffective or unsafe. However, identifying drugs that retain or restore visual function is challenging. Here, we demonstrate in a vertebrate model of inherited blindness that HDAC inhibitors and BDNF mimetics are sufficient to significantly improve cone-photoreceptor mediated visual function via a BDNF mediated signalling dependent mechanism of action.

Translating drug treatments for inherited blindness from research models to the clinic has been remarkably unsuccessful. In part, this reflects poor correlations between biochemical or morphological readouts of neuroprotection in research models and the more desirable clinical end-point of improved functional vision^41, 42^ Clinical setbacks can arise from trials on patients harbouring heterogenous genetic mutations that mask treatment efficacy, or poor ocular pharmacokinetics, resulting in optimal drug concentration of inefficient duration^43, 44^.

To enable efficient identification of drugs overcoming visual impairment, we applied the zebrafish *dye* model of inherited blindness coupled with established visual behaviour assays. Although *dye* is an imperfect model of retinal degeneration, it was chosen because *i*) the ~25% homozygous affected *dye* larvae can be reproducibly and easily phenotyped at 3 dpf; *ii*) *dye* larvae at 5 dpf exhibit pronounced visual behaviour and retinal morphology deficits, and *iii*) drug treatment of *dye* from 3–5 dpf followed by optokinetic or visualmotor response assays enable identification of small molecule drugs that prevent loss of visual function. The mutation in *dye* is caused by a 180 bp deletion in *atp6v0e1*, *a* vacuolar ATPase v0e1 subunit^45^, which affects splicing of *atp6v0e1* transcripts into mature mRNA. Vacuolar ATPase complexes acidify organelles via transportation of H^+^ ions across vesicle membranes^31^. In the retina, disruption of this complex leads to phagocytosis defects and accumulation of undigested photoreceptor outer segments in the RPE. Defective outer segment phagocytosis is a hallmark of retinal diseases including RP and Usher syndrome type IB^46–48^. N-retinylidene-N-retinylethanolamine (A2E), a major component of lipofuscin, is a potent inhibitor of v-ATPase activity and accumulation of lipofuscin is associated with age-related macular degeneration (AMD)^49^. In humans, mutations in V-ATPase a3 subunit cause osteopetrosis, wherein patients display visual impairment amongst other pathologies^50^. In the zebrafish eye, *atp6v0d1* expression is restricted to the outer nuclear layer (ONL) and inner nuclear layer (INL) of the retina at 2 dpf^39^, and to the RPE from 3 dpf. *atp6v0e1* shows a similar expression pattern from 3 dpf, where expression is mainly present in the RPE (Supplementary Fig. S1), Mutants of several other vacuolar ATPase subunits have similar retinal morphological defects to *dye* including RPE hypopigmentation, ciliary marginal zone cell death and abnormal photoreceptor morphology^39^. Defects in photoreceptor morphology are likely explained by aggregate accumulation in the RPE/photoreceptors, degradation of which is perturbed by an inability to acidify lysosomes. CMZ cell death affects a subpopulation of progenitor cells in the teleost retina^51, 52^ though it is currently unknown how the *dye* mutation impacts survival of these cells. Significantly, 5 dpf *dye* larvae recapitulate clinical features of human blindness including significantly attenuated vision. In *dye* electroretinograms, significantly diminished b-wave amplitudes are observed, indicating defective photoreceptor-induced depolarisation of bipolar cells. This is consistent with ERG recordings from alternative zebrafish models of blindness including mutations in other vacuolar ATPase subunits^39^, the *noir* mutant arising from defective pyruvate dehydrogenase metabolism^53^ and mutations of the SNARE complex, formation of which required for synaptic vesicle fusion^54^. Importantly, *dye* larvae exhibit impaired visual behaviour in optokinetic and visualmotor responses. These technically simpler and higher throughput assays are compatible with drug screening. In summary, the *dye* zebrafish model of inherited blindness is appropriate to incorporate into efficient phenotype-based screens to identify drugs that preserve or restore visual function.

In *dye*, HDACi treatment is sufficient to restore visual function. HDACi were chosen due to the contradictory reports in the literature regarding their efficacy and safety in clinical trials of patients with inherited sight loss and concerns over patients self-medicating with unapproved HDACi^22–25^. Significantly, HDACi rescues some features of retinal morphology, but robustly rescues visual responses. Previous studies report HDACi to preserve rod photoreceptor morphology *ex vivo* but this report demonstrates an improvement of visual function. In agreement, HDACi increased expression of phototransduction proteins including phosphodiesterase, opsin, transducin and peripherin isoforms. Despite the efficacy of HDACi in restoring visual function, translation to clinical intervention for patients with inherited sight loss is justifiably occluded due to ongoing safety and specificity concerns^22, 23^. Thus, we sought to identify alternative key signaling nodes that are sufficient to recapitulate the HDACi rescue.

Bioinformatic analysis unmasked Bdnf, Myc, MycN, Tp53 and RICTOR signaling as transcriptional regulators predicted to mediate the rescue of visual function by HDACi. Of the identified signalling mechanisms, the most notable was the correlation between visual rescue and BDNF signaling. BDNF is a neurotrophic growth factor whose mature form binds the tropomyosin related kinase B (TrkB) receptor. BDNF-TrkB signaling has diverse physiological functions, including regulation of photoreceptor development and maintenance^10^. For instance, TrkB null mice develop shortened photoreceptor outer segments^55^. In the adult rat retina, BDNF and TrkB co-localise in green-red-sensitive cone photoreceptor outer segments^56^. In developing zebrafish, Bdnf localisation is observed in the outer nuclear layer (ONL), outer plexiform layer (OPL) and inner plexiform layer (IPL), while TrkB localisation is restricted to the IPL^57^, suggesting that ganglion or Müller cells receive the BDNF signal in the retina. We speculate that cell signalling pathways activated by agonism of the TrkB receptor on ganglion or glial cells culminates in release of trophic factors that support photoreceptors, a mechanism reported in models of light-induced photoreceptor degeneration^58^.

*BDNF* transcription is known to be induced by HDACi treatment in cultured rat cortical neurons mediated by the CREB transcription factor^40^, and in combination with ciliary neurotrophic factor (CNTF), BDNF reduces cell death in *rd1* mouse retinal explants^15^. Furthermore, small molecule BDNF mimetics are neuroprotective in mammalian models of traumatic brain injury^59, 60^. Here, pharmacological approaches demonstrated the necessity and sufficiency of BDNF-TrkB signaling for visual rescue in *dye* larvae. ANA-12, a TrkB receptor antagonist blocked HDACi mediated rescue by 69% and 85% in OKR and VMR assays respectively. Critically, 7,8-dihydroxyflavone hydrate, a TrkB receptor agonist or BDNF mimetic is sufficient to restore visual function and reduce CMZ cell death in the *dye* model of inherited sight loss. Co-treatment with 7,8-DHF and ANA-12 blocked rescue of visual function (71% and ~54% decrease in OKR and VMR respectively, compared to 10 µM 7,8-DHF only treated larvae), with partial rescue of dying cells in the CMZ. Treatment appears to result in a decrease in the size of the CMZ, which is partially blocked by ANA-12 co-treatment. A summary of the signaling networks involved in HDACi and BDNF mimetic mediated rescue is presented in Supplementary Fig. S2. Our findings support the conclusion that BDNF-mediated retinal neuroprotection is conserved in zebrafish and mammalian models, highlighting the applicability of zebrafish as a model for translational neurodegeneration research.

BDNF mimetics offer an exciting alternative approach to treating sight loss. They offer a more selective approach than HDACi which have diverse target profiles and global effects on gene expression. BDNF was identified as a photoreceptor protectant over 20 years ago^12^ but translational studies delivering BDNF in biological forms were hampered by pharmacokinetic issues^61–63^. Small molecule BDNF mimetics can circumvent these issues as they: i) protect vision *in vivo*, ii) cross the blood-tissue barriers iii) can be delivered directly to the eye and iv) are more stable than BDNF. BDNF mimetics are well tolerated in mammalian models, demonstrate efficacy in rodent models of Alzheimer’s, Parkinson’s and Huntington’s disease and no adverse outcomes are reported for humans consuming 7,8 dihydroxyflavone as a nootropic supplement.

## Materials and Methods

### Ethics Statement

All experiments carried out on animals were performed according to ethical approval granted by the UCD Animal Research Ethics Committee. Zebrafish in the first 5 days of life are not capable of feeding independently. The UCD Policy on the use of Animals for Research & Teaching states that “Protected animals are those which have the capacity to experience pain, suffering, distress or lasting harm as a result of procedures which may be carried out in the course of research or teaching.” … “Larval stages of fish are judged to be capable of experiencing pain, suffering or distress once they are capable of feeding independently”. As zebrafish larvae under 5 days post fertilization were used in all experiments, no protected animals were used and experiments were not subject to full ethical review.

### N-nitroso-N-ethylurea (ENU) mutagenesis screen

Male zebrafish of the AB genetic background were treated for 1 hour with 3 mM ENU (Sigma) diluted in 10 mM sodium phosphate buffer, pH 6.6. This treatment was repeated 4 times at 7–14 day intervals. Treated males were mated and embryos were grown up to generate F_1_ founders outcrossed to wildtype fish generating F_2_ families. F_3_ offspring were screened for recessive mutations using the optokinetic response (OKR) assay. Carriers of mutations were crossed to wildtype Tubingen fish to generate hybrid carriers. These carriers were incrossed and their mutant and normal offspring were collected for phenotyping and genotyping.

### Identification of *dye* mutation

Genomic DNA was isolated from larval zebrafish using a DNeasy Blood and tissue kit (Qiagen) according to manufacturer’s instructions. DNA concentration was determined by spectrophotometry using a Nano-drop 2000 (Thermo Scientific). PCR utilised the Crimson-Taq DNA polymerase system (New England Biolabs). Extracted DNA was used for bulk segregation analysis using Z-markers as described in Sapetto-Rebow, *et al*.^64^. Within the genomic lesion, *atp6v0e1* was selected for further interrogation. Primers used for genotyping include: *atp6v0e1* A forward: AGAACCACTGCCAGAACC *atp6v0e1* A reverse: CGGTTCTGTAGAGCAGAAGA, *atp6v0e1* B forward: AGAGAGAAGAGGAAGAGCCA *atp6v0e1* B reverse: TAGAGCGGAACAGGAATCAC, *atp6v0e1* C forward: CCATCTGGTACCTGTACTACC, *atp6v0e1* C reverse: ATTTTGAGGAAACAACACCGAG, *atp6v0e1* D forward: TGACTTGACGCCTGAAGTATTA, *atp6v0e1* D reverse: AGTTCTCGTAGACCTGTTAGC, *atp6v0e1* E forward: GGAGGATGGTCCAGTAACAG *atp6v0e1* E reverse: GTGTCGGTACTGCTCTCAG, Exon 2-3 *atp6v0e1* forward: GCTGGTTCTGACTGCTGTCTGCT, exon 2-3 *atp6v0e1* reverse: GTGGCGACGGGTGTTCAGGG, *β-actin* forward: TGGCCCATCCATCGTTCACAGGA, *β-actin* reverse: CGCATCCTGAGTCAATGCGCCA. PCR products were run on a 1.2% agarose gel containing 0.2 μg/mL ethidium bromide and visualised using a Gene Genius Bioimaging System (Syngene). For identification of the *dye* deletion, PCR amplicons from pooled *dye* and WT larvae, generated using the *atp6v0e1* C primer pairs were extracted and purified using a GeneJet gel extraction kit (Fisher) according to the manufacturer’s instructions. DNA amplicons were sequenced on an Illumina platform (Source BioScience, Republic of Ireland). Alignments of the resulting sequence data were generated using MultAlin software^65^.

### RNA extraction and RT-PCR

RNA was extracted from 50 pooled whole larvae using Qiashredder columns (Qiagen, Germany) and an RNeasy Mini kit (Qiagen, Germany) in an RNase free environment. RNA samples were quantified using the Nanodrop ND-100 and RNA samples were reverse transcribed into cDNA using the Superscript III First-Strand Synthesis System (Invitrogen). Briefly, each RNA sample was mixed with 5 µM oligo(dT)primer and 1 mM dNTP mix in DEPC-treated water. Samples were incubated at 65 °C for 5 minutes and cooled on ice. A cDNA synthesis mix containing RT buffer, MgCl_2_, DTT, RNase OUT and Superscript III reverse transcriptase enzyme was added to each sample. Samples were incubated at 50 °C for 50 minutes and reactions were terminated at 85 °C for 5 minutes. Negative controls were synthesised in the same way without Superscript III enzyme. PCR was carried out on the resulting cDNA samples and amplification products were analysed on an agarose gel. Primers used for RT-PCR: *tcerg1* forward: AGCTCTACAGACGTCACTCCCCC, *tcerg1* reverse: TGTGTGTGTTCACGCAGCATCAGT, *atp6v0e1* forward: GCGATCGCGGTGATGACCCT and *atp6v0e1* reverse: GCAGACAGCAGTCAGAACCAGCA.

### Zebrafish maintenance

Heterozygous *dye* carriers were maintained as inbred stocks. Matings were carried out by transferring adult fish to mating tanks. Embryos were obtained by natural spawning and raised in petri-dishes containing standard embryo medium solution (0.137 M NaCl, 5.4 mM KCl, 5.5 mM Na_2_HPO_4_, 0.44 mM KH_2_PO_4_, 1.3 mM CaCl_2_, 1.0 mM MgSO_4_ and 4.2 mM NaHCO_3_, conductivity 1200 µs, pH 7.2) at 28.5 °C on a 14/10 hour light/dark cycle until 3 days post fertilization (dpf).

### Drug treatments

At 3 dpf *dye* larvae were separated from unaffected sibling larvae based on phenotype. *dye* mutants and siblings were treated with either 1 µM Trichostatin A (TSA, Sigma) with or without 100–500 nM ANA-12, 6 µM Scriptaid (Sigma), 10 µM MC1568 (Tocris Biosciences), 10 µM MS275 (Cayman Chemicals), 10 µM 7,8-dihydroxyflavone hydrate (Sigma) or 0.1% dimethyl sulfoxide (DMSO) vehicle control in a final concentration of 0.1% v/v DMSO/embryo medium solution. Optimum drug concentrations for treatments were determined by prior treatment with 0.25–4 µM TSA, 2-10 µM Scriptaid, 5–20 µM MC1568 and 5-20 µM MS275, 2.5–10 µM 7,8-dihydroxyflavone hydrate, and assessment of defects in gross morphology and optokinetic response. For each treatment 12 larvae were transferred to 10 mL drug solution in a 60 × 15 mm petri dish, sealed in a container, and incubated under standard conditions stated above until 5 dpf.

### Visual behaviour assays

At 5 dpf, HDACi-treated larvae were removed from drug solutions and transferred to embryo medium for behavioural analyses. In the optokinetic response (OKR) assay, individual larvae were transferred to a petri dish containing 9% methylcellulose for immobilisation, and placed inside a circular grated pattern comprising 18° black and white stripes. The pattern was rotated at 18 rpm for 30 seconds clockwise and 30 seconds anti-clockwise. The number of saccadian eye movements per minute was recorded manually. For the visual motor response (VMR) assay, treated larvae were transferred in 600 µL embryo medium to individual wells of a 96 well clear polystyrene plate (Whatman). The plate was placed in the Zebrabox® recording chamber (Viewpoint Life Sciences) and locomotor activity quantified in response to changing light conditions by a motion detecting infrared camera. Detection parameters and analysis was performed as described previously by Deeti, *et al*.^66^. All statistical analyses were performed in Graphpad Prism V6. A Kruskal-Wallis one-way analysis of variance with post-hoc Dunn’s multiple comparison test was performed for visual function data, comparing *dye* control larvae to all other groups unless otherwise stated^67^.

### Electroretinography

Larvae were dark adapted for 30 minutes and paralysed with 0.5 mg/mL mivacurium chloride (Mivacron), the dark-adapted status was maintained throughout the handling and measurement processes. The reference electrode was placed in E3 medium, the recording electrode was filled with 0.9% saline solution and positioned on the center of the cornea using a micromanipulator and amplified with a P55 pre-amplifier (Grass Instruments). A 300 W halogen light source was used for light stimulation. The maximum light intensity stimulus was 2.8 × 10^3^ µW/cm^2^ with a 20 ms flash duration at all intensities (controlled by a S48 stimulator (Grass Instruments). Three optical density filters produced flash intensities at −3.0, −2.0, −1.0 and −0 log of the maximum. Recordings were analysed as previously described^68^. Raw data from *dye* mutants and siblings was compared using a 2-sample t-test with unequal variances.

### Histological analysis

Samples were fixed overnight in 4% paraformaldehyde and 2.5% glutaraldehyde in 0.1 M Sorenson phosphate buffer at pH 7.3. For bright-field imaging, samples were washed in PBS and imaged using an Olympus SZX10 microscope. For light microscopy, samples were post-fixed in 1% osmium tetraoxide and dehydrated in gradient ascending series of ethanol concentrations prior to Epon 812 resin embedding overnight. 1 µm sections were prepared using a Leica EM UC6 microtome and glass knife, mounted on glass slides and stained with toluidine blue. Prepared sections were imaged by a Leica DMLB bright field illumination microscope and Leica DFC 480 camera. The number of dying cells in the ciliary marginal zone (CMZ) was quantified by recording pyknotic nuclei present in sections 5 µm apart surrounding the optic nerve. The area of the CMZ was measured in central retinal sections using the polygonal selection tool in imagej, morphology data was analysed using an ordinary one-way ANOVA. The area of the CMZ was determined according to the criteria in Raymond, *et al*.^69^. For transmission electron microscopy (TEM), 0.1 µm sections were prepared using a Leica EM UC6 microtome and diamond knife, transferred to a support grid, contrasted with uranyl acetate and lead citrate, and analysed on a FEI-Tecnai 12 BioTwin transmission electron microscope (FEI Electron Optics).

### Protein extraction and western blot

4 and 5 dpf HDACi-treated or control larvae were euthanised using 4% Tricaine, and transferred to protease inhibitor solution (cOmplete™, Mini Protease Inhibitor Cocktail, Sigma). Larvae eyes were enucleated. 50 larval eyes were sonicated at 5% amplitude for 10-15 s using a Soni-prep 150 (Sanyo) in 80 µL of either protease inhibitor solution or lysis buffer (50 mM HEPES/KOH pH 7.2, 5 mM EGTA, 10 mM KCl, 2 mM MgCl_2_, 0.1% CHAPS). Protein concentration was determined by Bicinchoninic Acid assay (Pierce). 20 µg protein extracts were prepared in SDS-sample buffer (10% v/v Glycerol, 7 mM SDS, 62.5 µM Tris-HCl pH 6.8, 75 µM bromophenol blue, 5 mM DTT) and boiled at 95 °C for 5 minutes. Samples were centrifuged at 14,000 RPM for 8 minutes to remove excess pigment and cell debris. Protein samples were separated on a 0.75 mm 12% Bis/Tris acrylamide SDS-PAGE resolving gel for 1 hour in running buffer (3.5 mM SDS, 25 mM Tris base, 0.2 M Glycine) and transferred to polyvinylidene fluoride membrane at 100 V for 1 hour in transfer buffer (25 mM Tris base, 0.2 M glycine) using a mini-PROTEAN 3 module and mini Trans-blot cell (Biorad). Membranes were blocked in 5% milk solution for 1 hour, and probed with primary antibodies for acetyl-Histone H3 (Lys9) (1:1000, Millipore #07-352), BDNF (1:500, Millipore, #AB1534), Trkb (1:500, Santa-Cruz, #sc-12), phospho-Akt (1:1000, Cell Signaling Technologies, #9275), Akt (1:1000, Cell Signaling Technologies, #9272) and β-actin (1:1000, Sigma, A5441) overnight. Following washes in PBS-T, membranes were incubated in horseradish peroxidase (HRP) conjugated secondary antibody (Goat anti-rabbit, Amersham, #NIF825; Goat anti-mouse, Amersham, #NA934VS) for 2–4 hours, washes repeated, and placed in enhanced chemiluminescence western blotting detection reagent (Amersham). HRP activity was recorded by a LAS-3000 bioluminescence imager. Densitometry was performed in ImageJ and relative optical-density was normalised to β-actin or total Akt expression. Western blot data was analysed in Graphpad Prism V6 using a two-tailed paired t-test.

### Proteomic analysis

Protein from 50 homogenised larval eyes was isolated with the addition of trichloroacetic acid (TCA, 20%), reduced using 200 mM dithiothreitol, alkylated with 200 mM iodoacetamide and digested overnight with trypsin (Sigma). Peptides were desalted with C18 STAGE tips^70^ and resuspended in 0.1% TFA. For mass spectrometry peptide fractions were analyzed on a quadrupole Orbitrap (Q-Exactive, Thermo Scientific) mass spectrometer equipped with a reversed-phase NanoLC UltiMate 3000 HPLC system (Thermo Scientific). Peptide samples were loaded onto C18 reversed phase columns (5 cm length, 75 µm inner diameter). Raw data from the Orbitrap Q-Exactive was processed using MaxQuant version 1.5.1.0^71^, incorporating the Andromeda search engine^71, 72^, incorporating the Andromeda search engine^72^. To identify peptides and proteins, MS/MS spectra were matched to the UniProt *Danio rerio* database. All searches were performed with tryptic specificity allowing two missed cleavages. The database searches were performed with carbamidomethyl (C) as fixed modification and acetylation (protein N terminus) and oxidation (M) as variable modifications. For the generation of label free quantitative (LFQ) ion intensities for protein profiles, signals of corresponding peptides in different nano-HPLC MS/MS runs were matched by MaxQuant applying a mass accuracy of at least 20 ppm and a maximum time window of 1 min^73^. Perseus statistical software (version 1.4.1.3) analysed the LFQ intensities^73^. Perseus statistical software (version 1.4.1.3) analysed the LFQ intensities^74^. Protein identifications were filtered to eliminate the identifications from the reverse database and common contaminants. Data was log transformed and t-test comparison of fractions carried out. For visualization using heat maps, missing values were imputed with values from a normal distribution and the dataset was normalized by z-score^75^. Gene ontology terms were identified and visualised by submitting identified gene lists to DAVID and PANTHER databases.

### Pathway analysis

Ingenuity Pathway analysis (IPA) was used to identify inferred transcriptional regulators (ITRs) of differentially expressed genes in the datasets. Statistical algorithms match each gene symbol, fold change value and ANOVA p-value to corresponding objects in the curated IPA knowledge database, which is then used to identify shared regulators of genes in the dataset and are assigned a score based on relevance to input genes. This analysis also infers activation status of upstream regulators based on abundance of downstream transcriptional targets. Top ITRs were included in bar-charts with the predicted activation status score. ITRs and their predicted activation status and western blot data was collated and used to generate an interaction network using STRING database.

## Electronic supplementary material

Supplementary Information

Supplementary data S2

Supplementary data S1
