# Supplementary material for: A Brain-Derived Neurotrophic Factor Mimetic Is Sufficient to Restore Cone Photoreceptor Visual Function in an Inherited Blindness Model
Source: Sci Rep. 2017 Sep 12;7:11320. doi: 10.1038/s41598-017-11513-5 (PMC5595969; doi:10.1038/s41598-017-11513-5)
Supplement: Supplementary file 1 — Supplementary Information [file 41598_2017_11513_MOESM1_ESM.docx]

**Supplementary information for**

**A Brain-Derived Neurotrophic Factor Mimetic Is Sufficient to Restore Cone Photoreceptor Visual Function in an Inherited Blindness Model.**

**Authors**: Conor Daly^1^, Lisa Shine^1^, Theresa Heffernan^1^, Sudhakar Deeti^1^, Alison L. Reynolds^1^, John J. O’Connor^1^, Eugène T. Dillon^1,2^, David J. Duffy^3,5^, Walter Kolch^1,3,4^, Gerard Cagney^1^ and Breandán N. Kennedy^1,*^.

**Affiliations**.^1^UCD School of Biomolecular & Biomedical Science, UCD Conway Institute, University College Dublin, Belfield, Dublin 4, Ireland.

^2^Food for Health Ireland, Science Centre South, University College Dublin, Belfield, Dublin 4, Ireland.

^3^Systems Biology Ireland, Science Link Building, University College Dublin, Belfield, Dublin 4, Ireland.

^4^School of Medicine, University College Dublin, Belfield, Dublin 4, Ireland.

^5^Current address: The Whitney Laboratory for Marine Bioscience & Sea Turtle Hospital, University of Florida, 9505 Ocean Shore Blvd. St. Augustine, FL 32080-8610, USA.

^*^Corresponding author

Email: [brendan.kennedy@ucd.ie](mailto:brendan.kennedy@ucd.ie)

Supplementary Figure S1 is related to Figure 1.

Supplementary Figure S2 is related to the discussion.

Supplementary Figure S3 is related to Figures 1, 3 and 5.

**
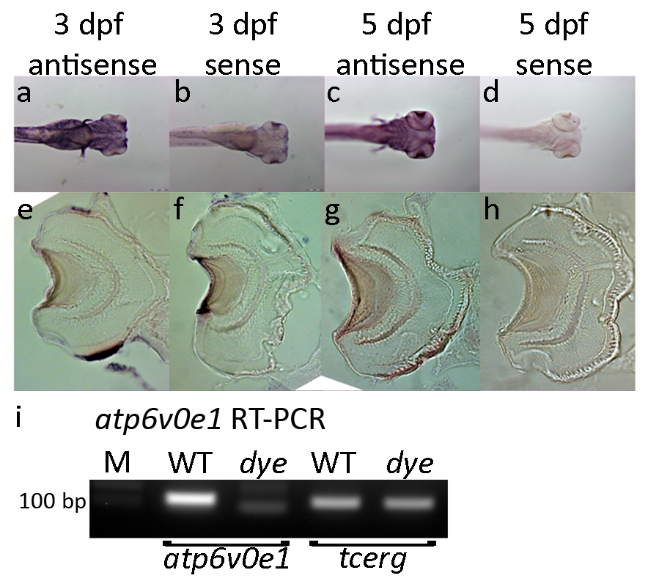
**

**Supplementary Figure S1: Expression pattern of *atp6v0e1* in wholemount and retinal sections of wildtype zebrafish larvae.**

(**a-h**) *In situ* hybridisation was carried out on 3 and 5 dpf wildtype larvae using antisense RNA probes to atp6v0e1 (n=3 for sense and antisense at each timepoint). In wholemount larvae (**a-d**), expression was detected in the brain and on the skin. At 3 and 5 dpf, expression is detected in the pectoral fins. The strongest expression levels were in the branchial arches at all timepoints (**a,c**). (**e-h**) 12 µm sections cut through the eyes of stained larvae revealed that expression was restricted to the dorsal RPE at 3 and 5 dpf (**e,g**), with higher expression levels at 3 dpf. **(i)** Agarose gel image for RT-PCR of atp6v0e1 and tcerg1, another candidate gene at the *dye* genetic locus. Expression of *atp6v0e1* mRNA is absent in dye, N=50 (number of larvae per replicate), n=3 (number of replicates).


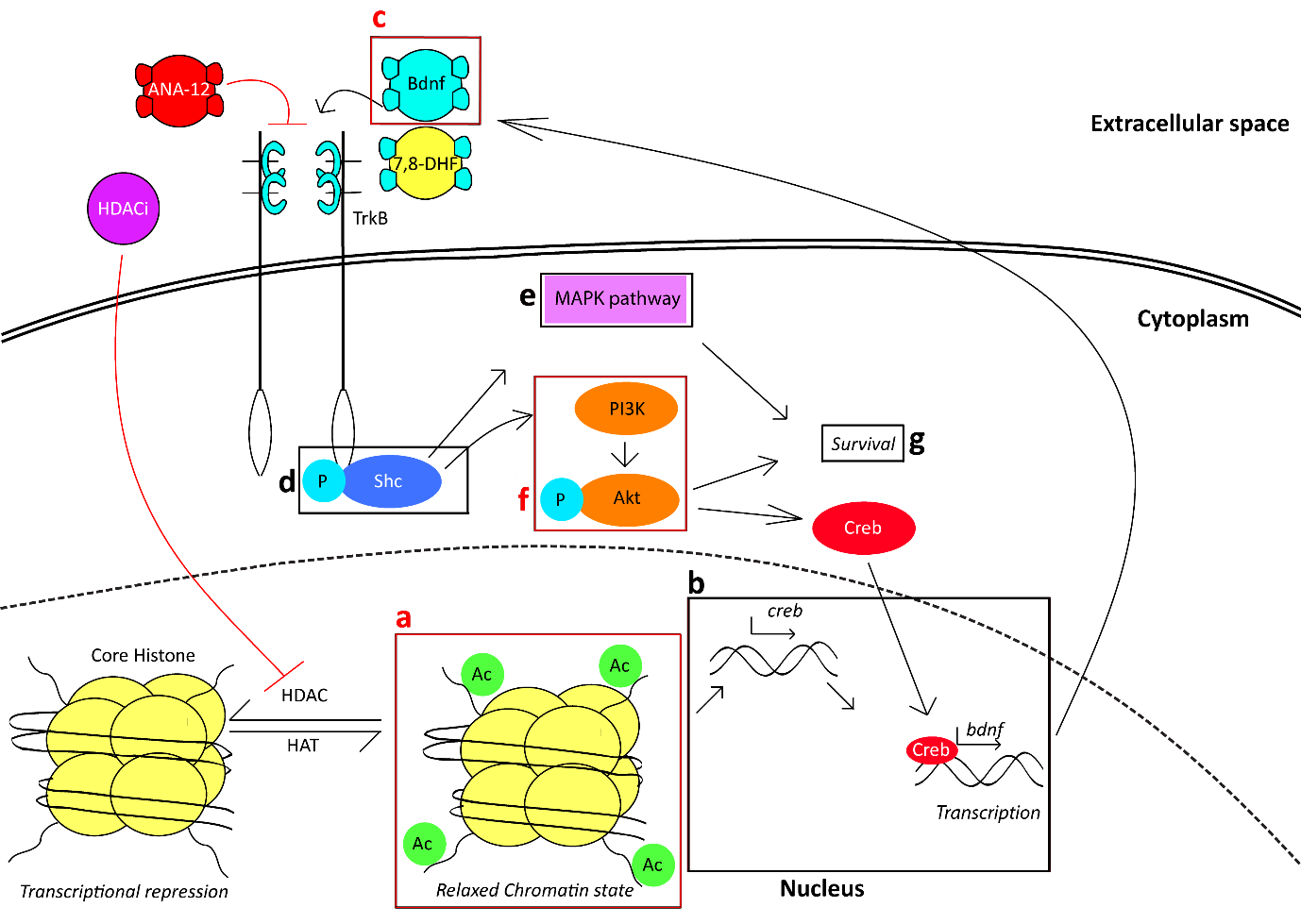


**Supplementary Figure S2: Schematic model of HDACi/BDNF mimetic mediated rescue in *dye*.**

(**a**) HDAC inhibition results in increased acetylation of core histones (demonstrated in **Fig. 3, e**), leading to a more relaxed chromatin conformational state and increased transcription of many genes including Bdnf via creb transcription factor (**b**, reference: Koppel and Timmusk ^1^, **c,** increased Bdnf expression demonstrated in **Fig. 5e**). Bdnf binds to the TrkB receptor in the extracellular space which leads to phosphorylation of Shc (**d**, reference: Kaplan and Miller ^2^) and other effectors resulting in activation of pathways involved in survival including MAPK (**e**, reference: Segal and Greenberg ^3^) and PI3K/Akt pathways (**f**, supported by evidence in **Fig** **5a,b** and **e**). In neurons, activation of the PI3K/Akt pathway can increase cell survival (**g**, reference: Dudek, et al. ^4^). Bdnf mediated rescue is attenuated by ANA-12, a pharmacological antagonist of the TrkB receptor. 7,8-dihydroxyflavone (7,8-DHF) treatment presents a more direct approach for targeting the BDNF-TrkB signaling pathway and restoring visual function.


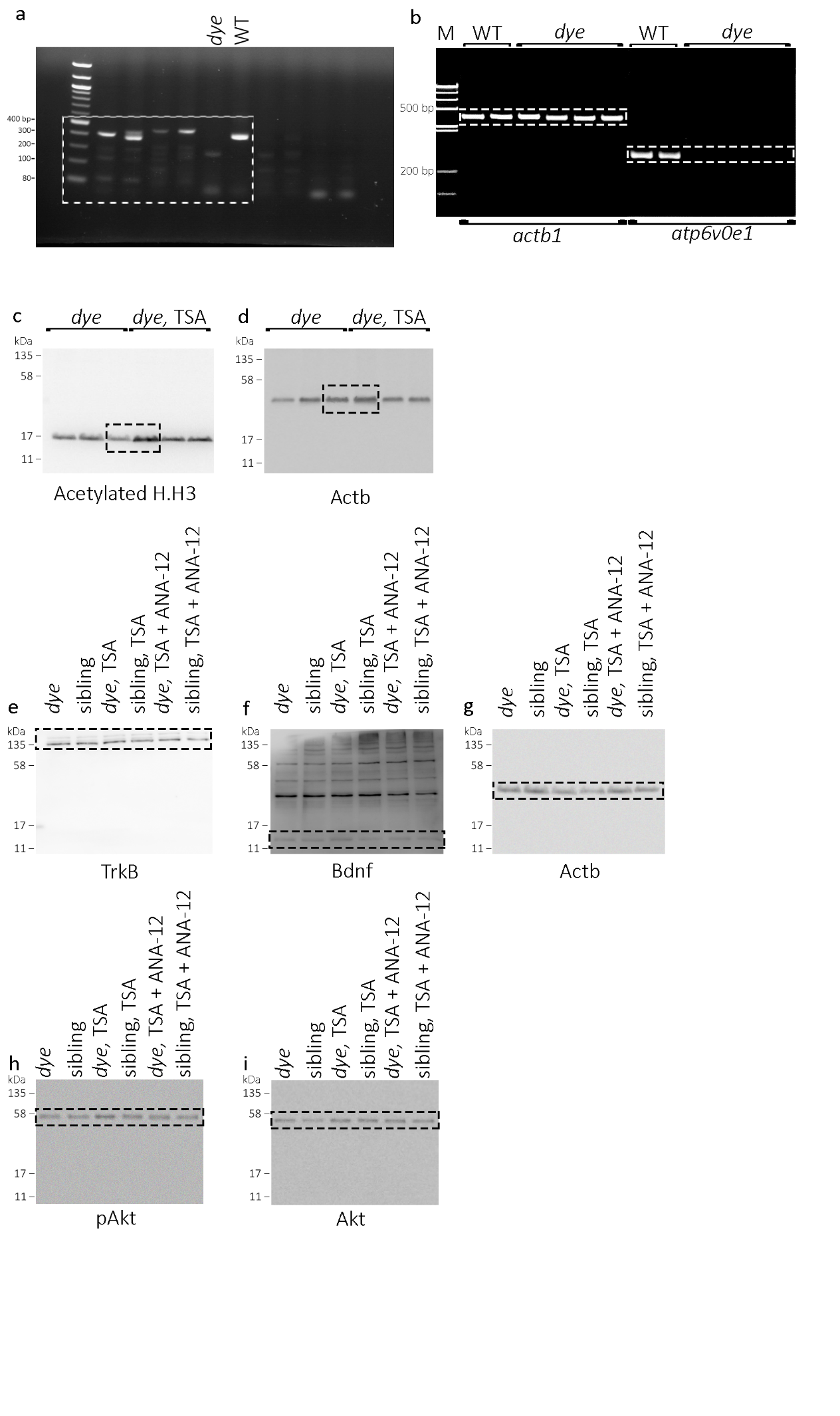
**Supplementary Figure S3: Agarose gels and western blots.**

(**a**) Agarose gel of PCR products used to identify the genomic region containing the deletion in *dye*. (**b**) PCR amplification of *actb1* (control) and *atp6v0e1*, in wildtype (WT) and *dye* larvae. (**c,d**) Acetylation status of histone H3 in response to TSA treatment in *dye* larvae. (**e-g)** Protein expression levels of TrkB (**e**), Bdnf (**f**), and loading control Actb (**g**). (**h,i**) phosphorylation status of Akt (at Thr308) (**h**) and control Akt (**i**)**.**

**References**

1 Koppel, I. & Timmusk, T. Differential regulation of Bdnf expression in cortical neurons by class-selective histone deacetylase inhibitors. *Neuropharmacology* **75**, 106-115, doi:10.1016/j.neuropharm.2013.07.015 (2013).

2 Kaplan, D. R. & Miller, F. D. Neurotrophin signal transduction in the nervous system. *Curr Opin Neurobiol* **10**, 381-391 (2000).

3 Segal, R. A. & Greenberg, M. E. Intracellular signaling pathways activated by neurotrophic factors. *Annu Rev Neurosci* **19**, 463-489, doi:10.1146/annurev.ne.19.030196.002335 (1996).

4 Dudek, H. *et al.* Regulation of neuronal survival by the serine-threonine protein kinase Akt. *Science* **275**, 661-665 (1997).
